# Draft genome sequence of *Marinobacterium rhizophilum* CL-YJ9^T^ (DSM 18822^T^), isolated from the rhizosphere of the coastal tidal-flat plant *Suaeda japonica*

**DOI:** 10.1186/s40793-017-0275-x

**Published:** 2017-10-30

**Authors:** Dong Han Choi, Gwang II Jang, Alla Lapidus, Alex Copeland, T. B. K. Reddy, Supratim Mukherjee, Marcel Huntemann, Neha Varghese, Natalia Ivanova, Manoj Pillay, Brian J. Tindall, Markus Göker, Tanja Woyke, Hans-Peter Klenk, Nikos C. Kyrpides, Byung Cheol Cho

**Affiliations:** 1 Marine Ecosystem and Biological Research Center, Korea Institute of Ocean Science and Technology, Ansan, 426-744 Republic of Korea; 2 Microbial Oceanography Laboratory, School of Earth and Environmental Sciences, and Research Institute of Oceanography, Seoul National University, Gwanak-ro, Gwanak-gu, Seoul, 151-742 Republic of Korea; 3 Centre for Algorithmic Biotechnology, St. Petersburg State University, St. Petersburg, Russia; 4 Department of Cytology and Histology, St. Petersburg Academic University, St. Petersburg, Russia; 5 Department of Energy Joint Genome Institute, Walnut Creek, CA USA; 6 Biological Data Management and Technology Center, Lawrence Berkeley National Laboratory, Berkeley, CA USA; 7 Leibniz Institute DSMZ – German Collection of Microorganisms and Cell Cultures, Braunschweig, Germany; 8 School of Biology, Newcastle University, Newcastle upon Tyne, UK

**Keywords:** Genome, *Marinobacterium rhizophilum*, *Suaeda Japonica*, Rhizosphere, GEBA

## Abstract

The genus *Marinobacterium* belongs to the family *Alteromonadaceae* within the class *Gammaproteobacteria* and was reported in 1997. Currently the genus *Marinobacterium* contains 16 species. *Marinobacterium rhizophilum* CL-YJ9^T^ was isolated from sediment associated with the roots of a plant growing in a tidal flat of Youngjong Island, Korea. The genome of the strain CL-YJ9^T^ was sequenced through the Genomic Encyclopedia of Type Strains, Phase I: KMG project. Here we report the main features of the draft genome of the strain. The 5,364,574 bp long draft genome consists of 58 scaffolds with 4762 protein-coding and 91 RNA genes. Based on the genomic analyses, the strain seems to adapt to osmotic changes by intracellular production as well as extracellular uptake of compatible solutes, such as ectoine and betaine. In addition, the strain has a number of genes to defense against oxygen stresses such as reactive oxygen species and hypoxia.

## Introduction

**Fig. 1:**
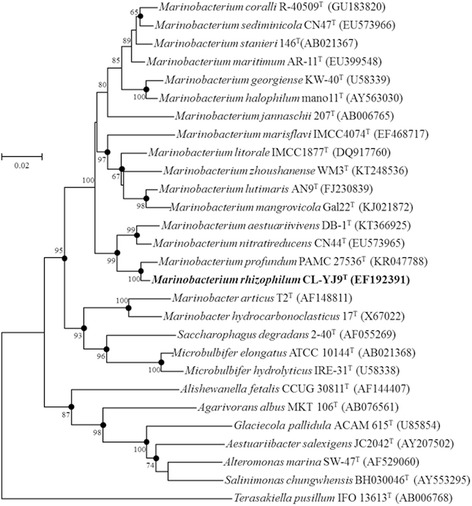
Neighbour-joining phylogenetic tree, based on 16S rRNA gene sequences, showing the relationships between strain CL-YJ9^T^, members of the genus *Marinobacterium* and other related genera. Bootstrap percentages >60% (based on 1000 resamplings) are shown below or above the corresponding branches. Solid circles indicate that the corresponding nodes are also recovered in the maximum-likelihood and maximum-parsimony trees. *Terasakiella pusillum* IFO 13613^T^ (AB006768) was used as an outgroup. Bar, 0.02 nucleotide substitutions per site

The genus 10.1601/nm.2860#_blank within the family 10.1601/nm.2805#_blank was established in 1997 by González et al. [1]. Currently the genus 10.1601/nm.2860#_blank contains 16 species with validly published names (Fig. 1). All 10.1601/nm.2860#_blank strains have been isolated from marine environments [1–11] such as sea water, tidal flat, deep-sea sediment, and coral mucus. Interestingly, their habitats include tropical waters [12, 13], Arctic marine sediment [7], tidal flats [4, 11] as well as deep sea sediment [10], indicating that the genus has well adapted to diverse environments. In the GOLD database [14], genome sequencing of 38 strains from 11 10.1601/nm.2860#_blank species are identified to be finished or in progress. In addition, six genome sequences from five species (10.1601/nm.2862#_blank, 10.1601/nm.11269#_blank, 10.1601/nm.13408#_blank, 10.1601/nm.2863#_blank and 10.1601/nm.28452#_blank) and one unidentified strain are found in the GenBank database. Among them, genomic features of 10.1601/nm.13408#_blank CL-YJ9^T^ (=10.1601/strainfinder?urlappend=%3Fid%3DDSM+18822#_blank=10.1601/strainfinder?urlappend=%3Fid%3DKCCM+42386#_blank ^T^), isolated from the rhizosphere of a plant *Suaeda japonica* inhabiting a coastal tidal flat, Korea, will be presented here.

## Organism information

### Classification and features

**Fig. 2:**
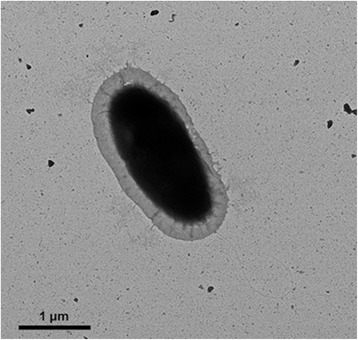
Transmission electron microscopy image of *Marinobacterium rhizophilum* CL-YJ9^T^

By phylogenetic analysis of the 16S rRNA gene sequence (Fig. 1), 10.1601/nm.13408#_blank strain CL-YJ9^T^ was positioned within the genus 10.1601/nm.2860#_blank and formed a distinct branch together with 10.1601/nm.28452#_blank 10.1601/strainfinder?urlappend=%3Fid%3DPAMC+27536#_blank ^T^ and 10.1601/nm.14295#_blank CN44^T^ (Fig. 1). Strain CL-YJ9^T^ was most closely related to 10.1601/nm.28452#_blank 10.1601/strainfinder?urlappend=%3Fid%3DPAMC+27536#_blank ^T^, which appeared as its sister species in the tree. Strain CL-YJ9^T^ grows under strictly aerobic conditions (Table 1). The optimal growth of strain CL-YJ9^T^ occurs at pH 7.0, with a growth range of pH 6.0–9.0. Growth occurs in the presence of 1.0–5.0% (*w*/*v*) NaCl (optimum 3.0%) and at 5–30 °C (optimum 25 °C) (Table 1). Cells of strain CL-YJ9^T^ are rod-shaped, on average approximately 0.3–0.4 μm wide and 0.6–0.8 μm long and motile by means of monopolar flagella (Fig. 2).

**Table 1:** Classification and general features of *M. rhizophilum* CL-YJ9^T^ [8, 9]

MIGS ID	Property	Term	Evidence code^a^
	Classification	Domain *Bacteria*	TAS [39]
		Phylum *Proteobacteria*	TAS [40]
		Class *Gammaproteobacteria*	TAS [41]
		Order *Alteromonadales*	TAS [42]
		Family *Alteromonadaceae*	TAS [43]
		Genus *Marinobacterium*	TAS [1]
		Species *Marinobacterium rhizophilum*	TAS [4]
		Type strain CL-YJ9^T^	TAS [4]
	Gram stain	Negative	TAS [4]
	Cell shape	Straight rods	TAS [4]
	Motility	Motile	TAS [4]
	Sporulation	Not reported	NAS
	Temperature range	5-30 °C	TAS [4]
	Optimum temperature	25 °C	TAS [4]
	pH range; Optimum	6.0-9.0; 7.0	TAS [4]
	Carbon source	Glucose, sucrose, mannose, glycerol, glycine, mannitol	TAS [4]
MIGS-6	Habitat	Sediment closely associated with the roots of a plant (*Suaeda japonica*)	TAS [4]
MIGS-6.3	Salinity	1-5% (optimum: 3%)	TAS [4]
MIGS-22	Oxygen requirement	Strictly aerobic	TAS [4]
MIGS-15	Biotic relationship	Microbiota of the rhizome of *Suaeda japonica*	TAS [4]
MIGS-14	Pathogenicity	Non-pathogenic	NAS
MIGS-4	Geographic location	Youngjong Island, Korea	TAS [4]
MIGS-5	Sample collection	November, 2005	TAS [4]
MIGS-4.1	Latitude	37.485^o^ N	TAS [4]
MIGS-4.2	Longitude	126.516^o^ E	TAS [4]
MIGS-4.3	Depth	Not reported	NAS
MIGS-4.4	Altitude	Not reported	NAS

^a^Evidence codes - *IDA* inferred from direct assay, *TAS* traceable author statement (i.e., a direct report exists in the literature), *NAS* non-traceable author statement (i.e., not directly observed for the living, isolated sample, but based on a generally accepted property for the species, or anecdotal evidence). These evidence codes are from the Gene Ontology project [44]

## Genome sequencing information

### Genome project history

The strain CL-YJ9^T^ was chosen for genome sequencing by the phylogeny-based selection [15, 16] as a part of the Genomic Encyclopedia of Type Strains, Phase I: the KMG project [17]. The KMG project, the first of the production phases of the GEBA: sequencing a myriad of type strains initiative [18, 19] and a Genomic Standards Consortium project [20] was set up to increase the sequencing coverage of key reference microbial genomes and to generate a large genomic basis for the discovery of genes encoding novel enzymes [21]. The genome sequencing, finishing and annotation were performed by the DOE-JGI using state of the art sequencing technology [22]. A summary of the project information is presented in Table 2.

**Table 2:** Genome sequencing project information

MIGS ID	Property	Term
MIGS-31	Finishing quality	Level 1: Standard Draft
MIGS-28	Libraries used	Illumina Std shotgun library
MIGS-29	Sequencing platforms	Illumina HiSeq 2000
MIGS-31.2	Fold coverage	119.1X
MIGS-30	Assemblers	Velvet v. 1.1.04, ALLPATHS v. R37654
MIGS-32	Gene calling method	Prodigal v2.5
	Locus Tag	F451
	Genbank ID	ARJM00000000
	Genbank Date of Release	12-Dec-2013
	GOLD ID	Gp0013985
	BIOPROJECT	PRJNA181367
MIGS-13	Source Material Identifier	CL-YJ9
	Project relevance	GEBA-KMG, Tree of Life

### Growth conditions and genomic DNA preparation

10.1601/nm.13408#_blank strain CL-YJ9^T^ was grown in DSMZ medium 514 (http://www.dsmz.de) at 28 °C and aerobe conditions. Genomic DNA was isolated using Jetflex Genomic DNA Purification Kit (GENOMED 600100) following the standard protocol provided by the manufacturer but additionally applying 50 μl proteinase K and using a 60 min incubation time. DNA is available through the DNA Bank Network [23].

### Genome sequencing and assembly

Using the purified genomic DNA, the draft genome of 10.1601/nm.13408#_blank CL-YJ9 ^T^ was generated at the DOE-JGI using the Illumina technology [24]. An Illumina standard shotgun library was constructed and sequenced using the Illumina HiSeq 2000 platform which generated 7,253,734 reads totaling 1088.1 Mbp. All general aspects of library construction and sequencing performed at the JGI can be found at the JGI website. All raw Illumina sequence data was passed through DUK, a filtering program developed at JGI, which removes known Illumina sequencing and library-preparation artifacts [25]. The following steps were then performed for assembly: (1) filtered Illumina reads were assembled using Velvet (version 1.1.04) [26], (2) 1–3 Kbp simulated paired end reads were created from Velvet contigs using wgsim (https://github.com/lh3/wgsim), (3) Illumina reads were assembled with simulated read pairs using Allpaths–LG (version r41043) [27]. Parameters for assembly steps were exactly same as in Choi et al. [28]. The final draft assembly contained 68 contigs in 58 scaffolds. The total size of the genome is 5.4 Mbp and the final assembly is based on 638.1 Mbp of Illumina data, which provides an average 119.1X coverage of the genome.

### Genome annotation

As described in Choi et al. [28], identification of genes was performed using Prodigal [29] as part of the DOE-JGI Annotation pipeline [30, 31]. After translation of the predicted CDSs, they were used to search the databases, such as National Center for Biotechnology Information non-redundant database, UniProt, TIGRFam, Pfam, PRIAM, KEGG, COG, and InterPro databases. Additional analysis and functional annotation were performed within the Integrated Microbial Genomes [32].

## Genome properties

The genome is 5,364,574 bp long and comprises 58 scaffolds ranging 1097 to 401,958 bp, with an overall G + C content of 58.5% (Table 3). Of the 4853 genes predicted, 4762 were protein coding genes, and 91 were RNA genes. A total of 3878 genes (79.9%) were assigned a putative function while the remaining ones were annotated as hypothetical or unknown proteins. The distribution of genes into COG functional categories is presented in Table 4. The properties and the statistics of the genome are summarized in Tables 3 and 4.

**Table 3:** Genome statistics

Attribute	Number	% of total^a^
Genome size (bp)	5,364,574	100
DNA coding (bp)	4,619,007	86.10
DNA G + C (bp)	3,136,815	58.47
DNA scaffolds	58	100
Total genes	4853	100
Protein coding genes	4762	98.12
RNA genes	91	1.88
Pseudo genes	0	
Genes in internal clusters	642	13.23
Genes with functional prediction	3878	79.91
Genes assigned to COGs	3433	70.74
Genes with Pfam domains	4066	83.78
Genes with signal peptides	386	7.95
Genes with transmembrane helices	1137	23.43
CRISPR repeats	1	

^a^The total is based on either the size of the genome in base pairs or the total number of protein coding genes in the annotated genome

**Table 4:** Number of genes associated with general COG functional categories

Code	Value	%age	Description
J	232	6.01	Translation, ribosomal structure and biogenesis
A	1	0.03	RNA processing and modification
K	289	7.48	Transcription
L	103	2.67	Replication, recombination and repair
B	2	0.05	Chromatin structure and dynamics
D	41	1.06	Cell cycle control, cell division, chromosome partitioning
V	72	1.86	Defense mechanisms
T	182	4.71	Signal transduction mechanisms
M	213	5.52	Cell wall/membrane/envelope biogenesis
N	71	1.84	Cell motility
U	58	1.50	Intracellular trafficking, secretion, and vesicular transport
O	162	4.19	Post-translational modification, protein turnover, chaperones
C	296	7.66	Energy production and conversion
G	334	8.65	Carbohydrate transport and metabolism
E	407	10.54	Amino acid transport and metabolism
F	102	2.64	Nucleotide transport and metabolism
H	211	5.46	Coenzyme transport and metabolism
I	179	4.63	Lipid transport and metabolism
P	186	4.82	Inorganic ion transport and metabolism
Q	134	3.47	Secondary metabolites biosynthesis, transport and catabolism
R	335	8.67	General function prediction only
S	209	5.41	Function unknown
–	1420	29.26	Not in COGs

The total is based on total number of protein coding genes in the annotated genome

## Insights from the genome sequence

To cope with osmotically varying conditions in tidal flat (e.g., exposure to heavy rainfalls or desiccation during low tides), 10.1601/nm.13408#_blank CL-YJ9^T^ seems to display diverse mechanisms of adaption. For instance, the strain can synthesize compatible solutes such as betaine, ectoine and 5-hydroxyectoine. The strain has two kind of genes (choline dehydrogenases and betaine aldehyde dehydrogenase; Table 5) participating in glycine-betaine biosynthesis from choline, which is found in Gram-negative bacteria [33]. The strain also has essential genes participating in the ectoine biosynthesis and the 5-hydroxyectoine biosynthesis (five enzymes for the steps from aspartate to ectoine as well as ectoine hydroxylase, respectively; Table 5) [34]. In addition, the strain seems to uptake osmolytes by transport from the external environment. In the genomic analysis, the glycine betaine/L-proline ABC transporter system known as proU, which is an operon that encodes a high-affinity ABC transporter system consisting of three proteins (ProV, ProW and ProX; F451DRAFT_00884, F451DRAFT_00885, F451DRAFT_00886, respectively) is found in the strain. Further, the homologue of the TRAP transporter (F451DRAFT_00922) involved in transport of external ectoine and hydroxyectoine is found in 10.1601/nm.13408#_blank. Function of the TRAP transporter is elucidated in both 10.1601/nm.2494#_blank 10.1601/strainfinder?urlappend=%3Fid%3DDSM+2581#_blank [35] and 10.1601/nm.1152#_blank 10.1601/strainfinder?urlappend=%3Fid%3DDSS+3#_blank [36]. Ectoine/5-hydroxyectoine-binding periplasmic protein in 10.1601/nm.13408#_blank showed amino acids sequence similarity of 35.1% and 33.8% with those of 10.1601/nm.2494#_blank (TeaA) and 10.1601/nm.1152#_blank (UehA), respectively. The transported ectoine is used as the sole carbon and nitrogen source in 10.1601/nm.1152#_blank, but 10.1601/nm.2494#_blank can use it as a compatible solute. Considering that ectoine can be de novo produced in 10.1601/nm.13408#_blank as well as actively transported from the environment, the role of the TRAP transporter in 10.1601/nm.13408#_blank could be thought to recover endogenously synthesized ectoine that has leaked through the membrane as known in 10.1601/nm.2494#_blank [35].

**Table 5:** Enzymes and gene-loci participating in selected pathways identified in the draft genome of *M. rhizophilum* CL-YJ9^T^. Gene-loci are from the IMG/MER database

Pathways	Enzymes	Gene-loci
Glycine betaine biosynthesis	Choline dehydrogenase	F451DRAFT_01661 F451DRAFT_03441 F451DRAFT_04658
	Betaine aldehyde dehydrogenase	F451DRAFT_00114
Ectoine and 5-hydroxyectoine biosynthesis	Aspartate kinase	F451DRAFT_00077 F451DRAFT_02577
	Aspartate semialdehyde dehydrogenase	F451DRAFT_01139 F451DRAFT_01140
	Diaminobutyrate aminotransferase apoenzyme	F451DRAFT_00080
	Diaminobutyrate acetyltransferase	F451DRAFT_00081
	Ectoine synthase	F451DRAFT_00079
	Ectoine hydroxylase	F451DRAFT_00078
Molybdopterin biosynthesis	Cyclic pyranopterin monophosphate synthase	F451DRAFT_03412 F451DRAFT_01249
	Molybdopterin synthase	F451DRAFT_04784 F451DRAFT_03411 F451DRAFT_01222

In the rhizosphere of tidal flat, oxygen tension varies in a wide range due to temperature change, repetitive exposure to atmosphere and seawater during tidal cycle and oxygen release from the roots of plants. Further, 10.1601/nm.13408#_blank has a molybdopterin biosynthesis pathway (Table 5) and molybdoenzymes that use molydopterin as cofactor or prosthetic group such as formate dehydrogenase (F451DRAFT_01667, F451DRAFT_01668, F451DRAFT_01669, F451DRAFT_01665) and arsenate reductase (F451DRAFT_01068). ROS can be generated during the molybdopterin metabolism. Thus, defense mechanisms to ROS are required. 10.1601/nm.2806#_blank sp. SN2, isolated from marine tidal flat, increased the number of oxidative stress tolerance genes to deal with ROS [37]. Similarly, many genes encoding ROS defense mechanisms are present in 10.1601/nm.13408#_blank, including catalase-peroxidae (F451DRAFT_01727, F451DRAFT_04596), superoxide dismutase (F451DRAFT_03202), alkyl hydroperoxide reductase (F451DRAFT_02876, F451DRAFT_01413, F451DRAFT_00847), glutathione peroxidase (F451DRAFT_01603) and glutaredoxin (F451DRAFT_00578, F451DRAFT_01573, F451DRAFT_04005) as direct ROS scavengers. This line of data indicates a lifestyle of 10.1601/nm.13408#_blank closely associated with the rhizosphere where substantial amounts of oxygen might be released from the roots of a well-adapted tidal-flat plant, *Suaeda japonica*. On the contrary, truncated bacterial hemoglobins (F451DRAFT_00578, F451DRAFT_01573, F451DRAFT_04005) involved in protection from oxidative stress and enhanced respiration under hypoxic conditions are present, indicating 10.1601/nm.13408#_blank is adapted to the hypoxic rhizosphere in tidal-flat sediments, too.

The presence of motility by means of monopolar flagella was reported in a previous report [4]. Consistently, a number of genes encoding flagellar basal body proteins, flagellar hook-associated proteins and flagellar biosynthesis proteins are found in the genomic analyses, suggesting that 10.1601/nm.13408#_blank could explore more favorable microenvironments using flagella in the rhizosphere. In contrast to a recent study that genes encoding steroid catabolism were identified in 10.1601/nm.2863#_blank S30 [38], most of these genes were not identified in the 10.1601/nm.13408#_blank.

## Conclusions

The genome of a representative of the genus 10.1601/nm.2860#_blank from the 10.1601/nm.808#_blank phylum is reported here for the first time. In addition to detailed information on genome sequencing and annotation, genetic adaptation in environmental conditions closely associated with rhizosphere of a tidal flat plant such as salinity change and oxygen stress could be understood on the basis of genomic analyses.

## Abbreviations

GEBA: Genomic Encyclopedia of *Bacteria* and *Archaea*
GOLD: Genomes OnLine Database
JGI: Joint Genome Institute
KMG: One thousand microbial genomes
ROS: Reactive oxygen species
TRAP: Tripartite ATP-independent periplasmic
